# Immersive virtual reality for interdisciplinary trauma management – initial evaluation of a training tool prototype

**DOI:** 10.1186/s12909-024-05764-w

**Published:** 2024-07-18

**Authors:** Laura Isabel Hanke, Lukas Vradelis, Christian Boedecker, Jan Griesinger, Tim Demare, Nicola Raphaele Lindemann, Florentine Huettl, Vuthea Chheang, Patrick Saalfeld, Nicolas Wachter, Jochen Wollstädter, Marike Spranz, Hauke Lang, Christian Hansen, Tobias Huber

**Affiliations:** 1 Department of General, Visceral and Transplant Surgery, University Medical Center Mainz, Johannes Gutenberg-University, Mainz Langenbeckstraße 1, 55131 Mainz, Germany; 2 Department of Anesthesiology, University Medical Center Mainz, Johannes Gutenberg-University, Mainz, Germany; 3 Virtual and Augmented Reality Group, Faculty of Computer Science, Otto-von-Guericke-University, Magdeburg, Germany; 4 Department of Orthopedics and Trauma Surgery, University Medical Center Mainz, Johannes Gutenberg-University, Mainz, Germany; 5 Department of Diagnostic and Interventional Radiology, University Medical Center Johannes Gutenberg-University, Mainz, Germany

**Keywords:** Virtual reality, Advanced trauma life support, Training, Interdisciplinary training, Immersive virtual reality training, Medical education, Trauma room

## Abstract

**Introduction:**

Emergency care of critically ill patients in the trauma room is an integral part of interdisciplinary work in hospitals. Live threatening injuries require swift diagnosis, prioritization, and treatment; thus, different medical specialties need to work together closely for optimal patient care. Training is essential to facilitate smooth performance. This study presents a training tool for familiarization with trauma room algorithms in immersive virtual reality (VR), and a first qualitative assessment.

**Materials and methods:**

An interdisciplinary team conceptualized two scenarios and filmed these in the trauma room of the University Medical Center Mainz, Germany in 3D-360°. This video content was used to create an immersive VR experience. Participants of the Department of Anesthesiology were included in the study, questionnaires were obtained and eye movement was recorded.

**Results:**

31 volunteers participated in the study, of which 10 (32,2%) had completed specialist training in anesthesiology. Participants reported a high rate of immersion (immersion(mean) = 6 out of 7) and low Visually Induced Motion Sickness (VIMS(mean) = 1,74 out of 20). Participants agreed that VR is a useful tool for medical education (mean = 1,26; 1 very useful, 7 not useful at all). Residents felt significantly more secure in the matter after training (*p* < 0,05), specialist showed no significant difference.

**Discussion:**

This study presents a novel tool for familiarization with trauma room procedures, which is especially helpful for less experienced residents. Training in VR was well accepted and may be a solution to enhance training in times of low resources for in person training.

**Supplementary Information:**

The online version contains supplementary material available at 10.1186/s12909-024-05764-w.

## Introduction

**Fig. 1:**
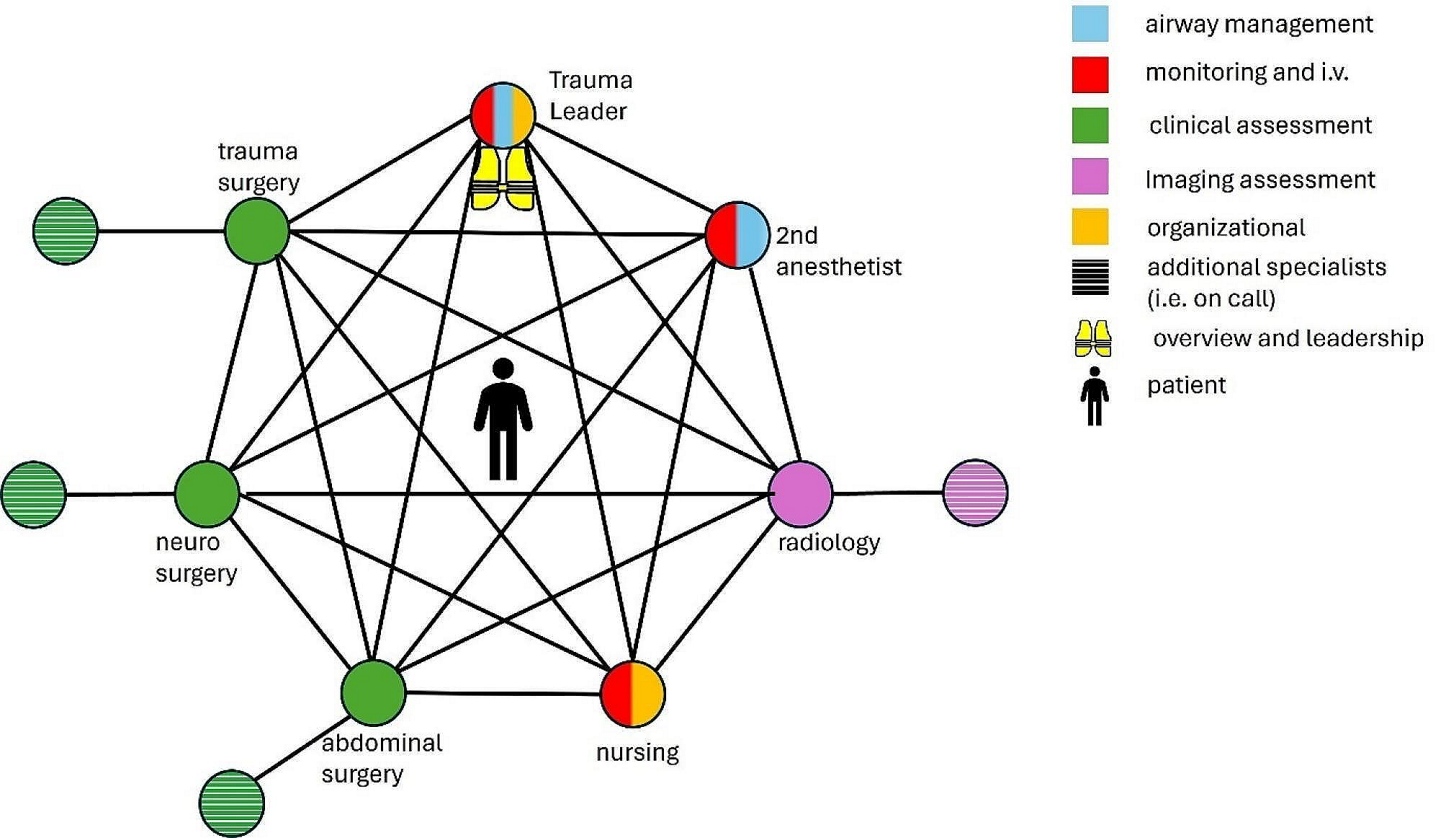
depiction of all members of the trauma team with their respective key roles. It should be noted that most team members are able to supplement or switch to different roles, however those are not their main duties. In the center of all is the patient

Emergency care of trauma patients is an integral part of in-hospital work. Trauma care in Germany is administered into different hospitals by their competency level and available specialties [1]. The University Medical Center Mainz, Germany is a level 1 trauma center and thus provides care for all types of injuries, amounting to about 1000 patients treated in the trauma room per year. The trauma room team consists of a core team including anesthesiology, radiology and orthopedic and trauma surgery and is extended depending on the patient’s needs. Every member of the team has specific tasks to fulfill in order to facilitate a swift and smooth execution of procedures in the trauma room [2]. Patients in the trauma room require fast diagnostics, interprofessional decisions and procedures because of their critical state. Figure 1 depicts the trauma team with their technical and nontechnical skills and tasks. The emergency trauma room resembles a well running machine or clockwork at its best. To accomplish this goal, regular training is essential [3–6]. Physicians are required to be proficient in their field and to be able to make life saving decisions and initiate and perform emergency procedures, which usually applies to physicians with completed specialist training or at the end of residency. It is recommended that each specialty and profession not only train on their own, but also as an interprofessional teams [7]. A recent international survey by Bento et al. has found potential for improvement on training opportunities [8]. Interprofessional trainings organized on a regular basis in the University Medical Center Mainz and is a precious resource in times of personnel shortages, thus only a handful of employees can train at a time. These trainings are elaborate and require regular hospital work to be reduced since the premises must be blocked off, and physicians, who would be needed in the OR need to be set free for the day to train. It has been common practice for younger residents to shadow during emergency trauma room procedures and to take on smaller parts of the procedure, such as assisting in documentation or patient transfer. Younger colleagues have mostly not taken part in an interdisciplinary trauma room training, since their availability is limited and usually reserved for advanced residents or early specialists. There is a written protocol to reference the procedure. Detecting a need for more training opportunities especially for younger residents, our group aimed to create a temporally and spatially flexible training resource to familiarize younger residents with the emergency trauma room setting and procedures. To be applicable for different specialties, novices and students we opted for a sole observer of an emergency trauma room procedure, without teaching of specific medical knowledge in the first prototype. To achieve our goal of a temporally and spatially flexible tool which is independent of trained instructors, and thus creating a resource-saving widely available tool, we chose immersive virtual reality (VR). Training in VR has been shown to be processed like autobiographical memories, which is thought to be beneficial for learning, in contrast to reading a text [9]. It has also been shown that training in VR is well received by different professions and age groups, making the learning experience more enjoyable [10, 11]. Therefore, we opted to evaluate subjective measures, such as self-perceived confidence in the topic as well as eye tracking as an objective measure. Eye tracking can indicate interaction with and perception of information from different sources in the virtual world [12]. Furthermore, this setting makes training times flexible and accessible for young residents as well as students, as no additional instructors or specific time slots or rooms are needed.

Previously, more realistic scenarios using real-life video material were preferred for training by participants over animated videos [13]. Similar projects have been described especially for military medical education [14–16] using simulated VR. This exploratory study aims to evaluate the first prototype of a fully immersive 360° VR trauma room training among anesthetists of different experience levels.

## Materials and methods

### Conceptualization of scenarios and video shoot

An interdisciplinary team of persons responsible for the trauma team training in the different departments conceived and scripted two scenarios. In both scenarios, the trauma leader explains his work and the processes of the trauma room to a medical student. When the patient arrives, clear communication between all team members is key and is depicted, enabling the viewer to perceive each action. The general goal of the VR application was to supplement trauma room experience to medical professionals. The aim of this first evaluation described was acceptance of VR training methods and differences among the users depending on their prior experience and personal characteristics.

#### Scenario 1

A young man riding a bicycle was involved in a traffic accident and brought to the trauma team by an emergency physician. The patient is awake and cardiorespiratory stable on arrival, he has no visible wounds and claims to have abdominal pain. In the trauma diagnostics No injuries were found in the trauma diagnostics.

#### Scenario 2

An elderly patient is brought in by an emergency doctor with reduced vigilance and compromised blood pressure but no known trauma. In the focused assessment with sonography for trauma (FAST), large amounts of free intra-abdominal fluid are found. A CT scan shows a ruptured spleen, and the patient is brought to angiography for further treatment.

A detailed script of the scenarios is provided as a supplement (Supplement 3).

In both scenarios, the role of each team member is clearly depicted and their tasks are emphasized. A total of ten physicians, three nurses and one medical student are involved in both scenarios, an actor portrays the patient. The videos were filmed using a 3D 360° camera in 8k (Insta360 Pro 2; Insta360, Guangdong, China) with the support of a company specialized in 360° video shooting (Visual-Impressions GmbH, Magdeburg, Germany). Filming took place in the actual trauma room and the Department of Radiology and included real medical equipment. Each scenario is about 35 min long.

### Creation of the 360° immersive virtual reality environment

The 360° videos obtained were edited and stitched based on the scenarios using Shotcut video editing tool (Meltytech, LLC, United States). We utilized the cross-platform game engine Unity (version 2019.2.18f1) as an environment for VR development. A Virtual Reality Toolkit (VRTK) was used to develop basic VR interactions. The VR environment with 360° videos was developed in a manner similar to the that described in [18]. We used the VIVE Media Decoder, a high-performance video decoding plugin, to implement video streaming in VR. For eye tracking, VIVE SRanipal and Tobii XR SDKs were used to access the eye tracking capabilities of HTC Vive Pro Eye VR headset in Unity. This VR headset provides 110° trackable field of view with 120 Hz tracking gaze data output frequency and 0.5°–1.1° accuracy for eye tracking performance. A heat map was generated using tracked gate data that visualizes the points of interest during the session. A mapping layer with the heat map texture was developed to track the gaze data and generate the heat map in real time. Additionally, recorded gaze data and timestamps can be applied to create a replay video with real-time gaze and heat-map visualizations. We used FB Capture SDK (version 2.25) and its metadata injection to record the replay 360° video while the user is performing in VR. This allows post-hoc assessment and scoring. Patel et al. [19] described their process of creating an immersive 360° VR environment, which differs from our set-up, but may be helpful for other centers seeking to create their own applications.

### Recruiting and data collection

**Fig. 2:**
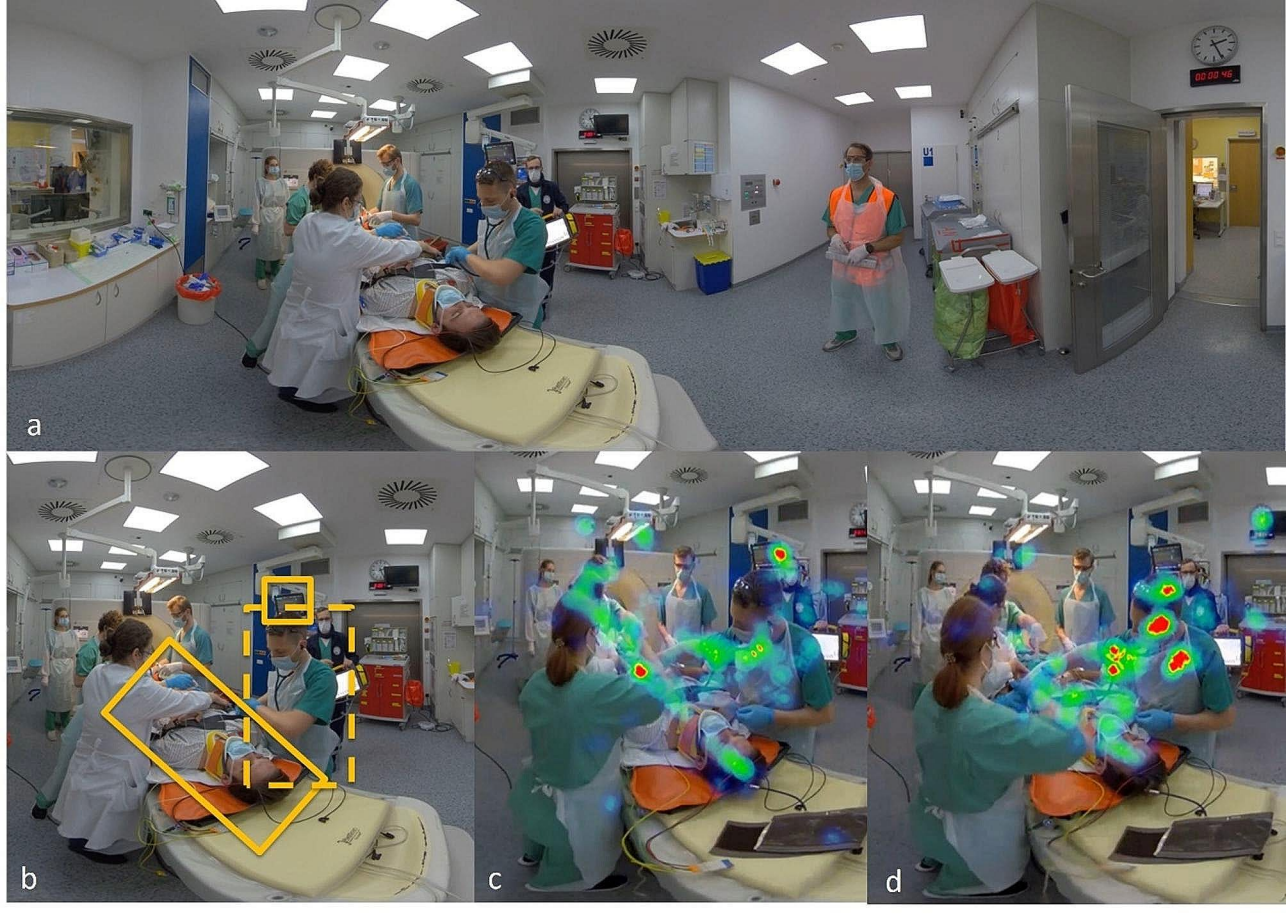
**a** shows a scene of the training scenario in immersive virtual reality, the patient has been transferred to the CT, different medical professionals are working on the patient, while the trauma leader on the right supervises them. In **b regions of interest (**ROIs) are defined by yellow squares, in this part of the scenario ROIs are the patient, the second anesthesiologist and the monitor. **c** shows the heat map of an experienced anesthetist (13 years of work experience) and **d** the heatmap of an anesthetist in his first months of his first year. While the experienced anesthetist watches the patient, the monitor and the examiner, the young colleague appears to mainly watch the examiner. This may be due to the inexperienced anesthetist trying to learn from the actions of the examiner. This effect, while interesting did not show a significant difference in eye tracking overall

The study collective is comprised of volunteering physicians working in the Department of Anesthesiology of the University Medical Center Mainz. Informed consent was obtained from all participants. Potential participants were informed about the study via regularly repeated e-mails as well as on a personal basis. The subjects watched both videos wearing a VR headmounted-display (HTC Vive Pro Eye, Taoyuan, Taiwan). Before starting the application, a short introduction into the study and VR equipment as well as the possibility of motion sickness, was provided. The head mounted display was calibrated for the subject before use, utilizing the Eye Tracking Calibration of the Steam VR dashboard. Eye Tracking was recorded as a separate data file as well as video file. The results are displayed as colored heat maps. A brief look only turns the track blue, a longer observation will turn the heat map yellow, and finally red. An example of this is shown in Fig. 2. Subjects were instructed to follow the video attentively and were blinded to the use of eye tracking. Following the scenarios, each subject completed a short questionnaire that consisted of personalized data, the Visual Induced Motion Sickness Scale (VIMS Scale) [17], the Immersion Scale by Nichols [18] and questions to subjective knowledge regarding trauma care and perception of beneficing of VR equipment in medical education and training using a 7 point Likert scale.

Subjective knowledge of procedures in the emergency trauma room were assessed using a 7-point Likert scale and compared between physicians who had completed specialist training in anesthesiology and residents using ANOVA. Subjects were asked to assess their knowledge prior to VR training and past VR training.

### Statistical analysis

The participants’ characteristics were analyzed using descriptive parameters. Further questionnaire items were analyzed using Chi-square and ANOVA test and grouping for different parameters. Statistical analysis was performed using SPSS 23 (SPSS, IBM, Armon, NY, USA). Eye Tracking was only analyzed in video 1. For analysis, regions of interest (ROIs) were defined, and two physicians of the study group (LH and LV) rated if participants looked at ROIs briefly [1], extensively [2], or not at all (0) by assessing the colors. Both raters were blinded to the identity and experience of participants and only worked by identification numbers. Raters scores were added and analyzed using Mann-Whitney-U test. An example of an eye tracking heat map and ROIs is depicted in Fig. 2.

## Results

A total of 31 volunteers of the Department of Anesthesiology of the University Medical Center Mainz were included. Ten (32.2%) participants had already completed specialist training in anesthesiology, 14 (45.2%) had finished emergency medical training and were working as emergency physicians. The other participants had not finished 3 years of specialist training, yet. The University Medical Center Mainz organizes emergency trauma room trainings on a regular basis. Of the participants 13 (41.9%) had attended this training or a similar one in the past. Detailed participants’ characteristics are displayed in Table 1. Participants were further analyzed by their experience with VR equipment (see also supplement 4). While mean age did not differ significantly between groups, there was a significant difference in gender distribution with more male participants with VR experience (*p* = 0.032).

**Table 1:** Participants characteristics including personal and professional features

Parameter	*n*
n	31
m / f / d	18 / 13 / 0
Age (median, min.; max. in years)	33 (26; 58)
Specialist training completed (y / n)	10 / 21
Years working in anesthesiology (median, min.; max.)	4 (1; 25)
Emergency medicine training completed (y / n)	14 / 17
PGY 3 or younger	14
Prior experience with VR technology (none / less than 5 times / more than 5 times / more than 10 times)	19 / 9 / 1 / 2
Visual aid (y / n)	19 / 12
Visual aid used (y / n)	13 / 6
Emergency trauma room training completed in the past (y / n)	13 / 18

Mean VIMS was 1.74 with a standard deviation of 3.098 and a minimum of 0 and maximum of 13. None of the participants had to interrupt or discontinue the training because of VIMS or any other reason.

**Fig. 3:**
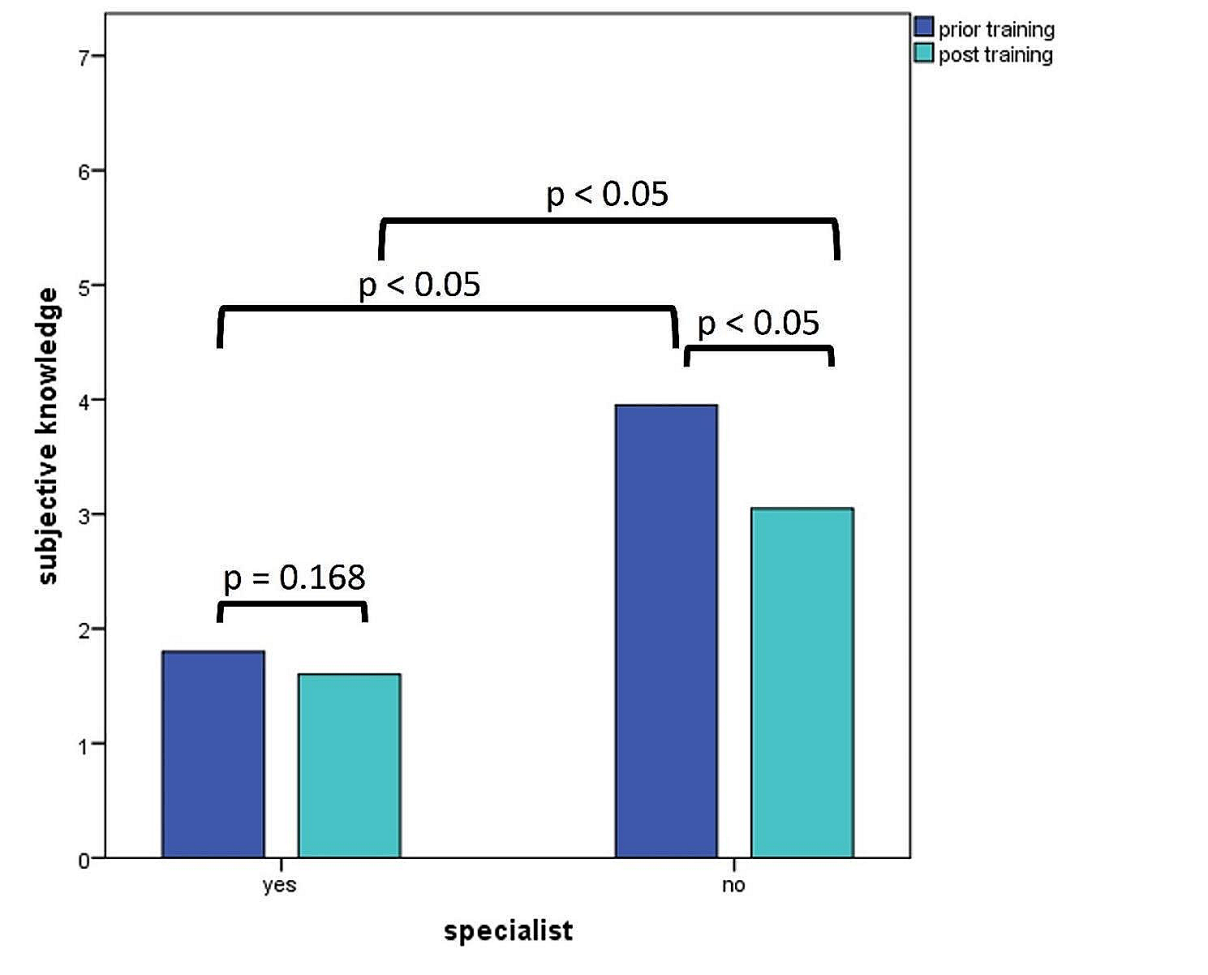
Participants were asked how secure they felt concerning emergency trauma room procedures prior and after training, 1 was “very secure” while 7 was “very insecure”. Specialists felt very secure before and after training, which is likely to be attributed to their long-term work experience. Residents were significantly less secure than specialists (p(prior) < 0.05; p(post) < 0.05) and felt significantly more secure after training (*p* < 0.05)

Specialists felt very secure about the procedures prior and after VR training. Residents reported to feel unsure prior to VR training and rather secure after. Both prior and after VR training there was a significant difference in subjective knowledge between specialists and residents (p_prior_ < 0.05; p_post_ = 0.002). Specialists showed no significant change in subjective knowledge (*p* = 0.168), while residents felt significantly more secure in the matter after VR training (*p* < 0.05). Figure 3 depicts the details of these results.

Participants reported to feel like they were present in the emergency trauma room, reaching a mean of 6 out of 7 (1 - not at all to 7 - very much). Furthermore, they were asked if they felt VR to be a useful tool in medical education on a 7-point Likert scale (1 - very useful to 7 - not useful at all). Subjects regarded the use of VR in medical education as very useful (mean 1.26), specialists and residents agreed on this matter (*p* = 0.724).

Eye Tracking was evaluated in 25 of 31 participants due to technical difficulties in recording of eye movements in 6 participants. Participants were divided by “specialist training in anesthesiology completed (yes / no)”. There was a significant difference in the observation of the emergency physician during patient hand-over, with residents watching the emergency doctor for a longer time (*p* = 0.008). For all other ROIs there was no significant difference between residents and specialists. To further explore the different groups Mann-Whitney-U Test was also performed using “emergency medicine qualification obtained (yes / no)” and “participated in an emergency trauma room training in the past (yes / no)” as grouping variables. There were no significant differences in the observed ROIs in these tests (*P* = 0.695 and *P* = 0.734, respectively). Interobserver reliability was high (Cronbach’s Alpha = 0.95).

## Discussion

We introduce an innovative tool for familiarization and training in emergency trauma rooms, which received positive feedback from participants. This novel training method was feasible even during the COVID-19 pandemic, which acted as a catalyst for the project’s initiation. Additionally, other groups demonstrated that virtual reality (VR) could supplement existing training programs and courses, effectively substituting certain components even during periods of social distancing [19, 20]. Furthermore, training sessions can be conducted at flexible hours and on short notice, such as during extended lunch breaks or at the end of regular shifts. Participants reported gaining a more comprehensive understanding of emergency trauma room procedures. While typically focused on their specific tasks within the trauma team, they gained greater insight into the overall process and the responsibilities of other team members. This was particularly beneficial for PGY1 residents, who were eager to train before their first night shifts. Eye-tracking data indicated a tendency for younger residents to observe the second anesthetist working on the patient, likely due to their future role in the trauma team (see Fig. 2). However, this finding was not statistically significant. The only significant difference observed in eye tracking was during patient handover, where younger residents focused on the emergency physician. This behavior suggests that more experienced anesthetists, accustomed to the trauma leader role, listen to the handover while monitoring the overall situation, whereas younger residents focus on the emergency physician to avoid missing any information. This is consistent with previous studies showing that experienced surgeons can engage in multitasking, such as taking phone calls, without impairing their surgical performance, unlike their less experienced counterparts. [21].

The immersive VR training was met with curiosity and high interest, with overwhelmingly positive feedback from participants. However, there are several limitations to the current program. The application is tailored specifically to the procedures and facilities of the University Medical Center Mainz, making it difficult to adapt for use in other institutions. Currently, the application simulates the role of an independent observer in the emergency trauma room and does not support interactive capabilities. Consequently, while it is suitable for familiarizing all medical specialties and professions with emergency trauma room procedures, it does not provide in-depth training on specific knowledge or medical procedures in its initial prototype. Future developments will include flow charts of responsibilities and educational slides within the VR application. Additionally, training in decision-making and task simulations will be incorporated to benefit more advanced physicians. Similar applications using animated simulations have been described previously and are partially available commercially [14, 16]. We agree with Couperus et al., who have presented a prototype of a military application for trauma training in simulated VR, that these types of simulations can create systems comparable to fight simulation training [14]. However, these advanced applications may not be as suitable for first-time employees or medical students, as they are more specialized for different medical fields. Currently, there is an educational gap in procedural knowledge and organizational understanding among novice physicians that we aim to address. The current program was well received and provides a foundation for future expansions tailored to various specialties and experience levels.

Optimal trauma room care requires training not only in medical knowledge and procedures but also in communication and leadership skills. For this reason, our hospital incorporates real-life training that includes serious games conducted in larger groups. Prior studies [3, 22] have underscored the importance of such training methods, independent of VR usage. The current program allows users to observe optimal communication practices, as noted by several participants, but it does not provide direct instruction or training in communication skills. Effective communication training requires group participation to enable practical exercises. While VR can enhance communication team training, our focus was on its capability to provide flexible, individual training without the need for training partners and instructors.

This study presents an initial user evaluation of a prototype for a new custom educational tool in immersive VR, and thus, the number of participants is limited. Future goals include implementing this tool for new and first-time employees, as well as further evaluating and developing interactive solutions.

### Electronic supplementary material

Below is the link to the electronic supplementary material.

Supplementary Material 1

## Data Availability

Data are available from the corresponding author upon reasonable request.

## Declarations

### Summary statement

Interdisciplinary training is key for care of critically ill trauma patients. We present a prototype of a trainings tool in immersive virtual reality with high acceptance and significant subjective knowledge improvement.

## References

[1] Ketter V, Ruchholtz S, Frink M. [Trauma center management]. Med Klin Intensivmed Notfmed. 2021;116(5):400–4. 33847765 10.1007/s00063-021-00807-2

[2] Tiel Groenestege-Kreb D, van Maarseveen O, Leenen L. Trauma team. Br J Anaesth. 2014;113(2):258–65. 24980423 10.1093/bja/aeu236

[3] Fernandez R, Rosenman ED, Olenick J, Misisco A, Brolliar SM, Chipman AK, et al. Simulation-based Team Leadership Training improves Team Leadership during actual trauma resuscitations: a Randomized Controlled Trial. Crit Care Med. 2020;48(1):73–82. 31725441 10.1097/CCM.0000000000004077

[4] Hong Y, Cai X. Effect of team training on efficiency of trauma care in a Chinese hospital. J Int Med Res. 2018;46(1):357–67. 28661260 10.1177/0300060517717401 PMC6011287

[5] Happel O, Papenfuss T, Kranke P. [Training for real: simulation, team-training and communication to improve trauma management]. Anasthesiol Intensivmed Notfallmed Schmerzther. 2010;45(6):408–15. 20539968 10.1055/s-0030-1255348

[6] Aggarwal R, Mytton OT, Derbrew M, Hananel D, Heydenburg M, Issenberg B, et al. Training and simulation for patient safety. Qual Saf Health Care. 2010;19(Suppl 2):i34–43. 20693215 10.1136/qshc.2009.038562

[7] McLaughlin C, Barry W, Barin E, Kysh L, Auerbach MA, Upperman JS, et al. Multidisciplinary Simulation-based Team Training for Trauma Resuscitation: a scoping review. J Surg Educ. 2019;76(6):1669–80. 31105006 10.1016/j.jsurg.2019.05.002

[8] Bento A, Ferreira L, Yánez Benitez C, Koleda P, Fraga GP, Kozera P, et al. Worldwide snapshot of trauma team structure and training: an international survey. Eur J Trauma Emerg Surg. 2023;49(4):1771–81. 36414695 10.1007/s00068-022-02166-9

[9] Schöne B, Wessels M, Gruber T. Experiences in virtual reality: a window to autobiographical memory. Curr Psychol. 2019;106:1–5.

[10] Barteit S, Lanfermann L, Bärnighausen T, Neuhann F, Beiersmann C. Augmented, mixed, and virtual reality-based head-mounted devices for Medical Education: systematic review. JMIR Serious Games. 2021;9(3):e29080. 34255668 10.2196/29080 PMC8299342

[11] Behmadi S, Asadi F, Okhovati M, Ershad Sarabi R. Virtual reality-based medical education versus lecture-based method in teaching start triage lessons in emergency medical students: virtual reality in medical education. J Adv Med Educ Prof. 2022;10(1):48–53. 34981005 10.30476/JAMP.2021.89269.1370 PMC8720154

[12] Clay V, König P, König S. Eye Tracking in virtual reality. J Eye Mov Res. 2019;12(1). 10.16910/jemr.12.1.3 PMC7903250 33828721

[13] Huber T, Paschold M, Hansen C, Lang H, Kneist W. Artificial Versus Video-based immersive virtual surroundings: analysis of performance and user’s preference. Surg Innov. 2018;25(3):280–5. 29504470 10.1177/1553350618761756

[14] Couperus K, Young S, Walsh R, Kang C, Skinner C, Essendrop R, et al. Immersive virtual reality Medical Simulation: Autonomous Trauma Training Simulator. Cureus. 2020;12(5):e8062. 32542120 10.7759/cureus.8062 PMC7290117

[15] Lombardo R, Walther N, Young S, Gorbatkin C, Sletten Z, Kang C, et al. Ready Medic one: a feasibility study of a semi-autonomous virtual reality Trauma Simulator. Front Virtual Real. 2022. 10.3389/frvir.2021.719656;2. 10.3389/frvir.2021.719656;2

[16] Colonna AL, Robbins R, Stefanucci J, Creem-Regeh S, Patterson B, Engel BT, et al. Trauma Bay virtual reality - A game changer for ATLS instruction and Assessment. J Trauma Acute Care Surg. 2022. 10.1097/TA.0000000000003569. 10.1097/TA.0000000000003569 35170584

[17] Keshavarz B, Hecht H. Validating an efficient method to quantify motion sickness. Hum Factors. 2011;53(4):415–26. 21901938 10.1177/0018720811403736

[18] Nichols S, Haldane C, Wilson JR. Measurement of presence and its consequences in virtual environments. Int J Hum Comput Stud. 2000;52(3):471–91. 10.1006/ijhc.1999.0343

[19] Johnson G, Beaumont J, Paton-Gay JD, Widder S, Gillman LM. Multidisciplinary, multisite trauma team training during COVID-19: lessons from the first virtual E-S.T.A.R.T.T. course. Can J Surg. 2021;64(6):E609–12. 34759046 10.1503/cjs.009921 PMC8592779

[20] Sinou N, Sinou N, Filippou D. Virtual reality and augmented reality in anatomy education during COVID-19 pandemic. Cureus. 2023;15(2):e35170. 36949987 10.7759/cureus.35170 PMC10029107

[21] Hsu KE, Man FY, Gizicki RA, Feldman LS, Fried GM. Experienced surgeons can do more than one thing at a time: effect of distraction on performance of a simple laparoscopic and cognitive task by experienced and novice surgeons. Surg Endosc. 2008;22(1):196–201. 17705087 10.1007/s00464-007-9452-0

[22] Paige J, Garbee D, Yu Q, Kiselov V, Rusnak V, Detiege P. Moving along: Team Training for Emergency Room Trauma transfers (T(2)ERT(2)). J Surg Educ. 2019;76(5):1402–12. 30987920 10.1016/j.jsurg.2019.03.013

