# Supplementary material for: Immersive virtual reality for interdisciplinary trauma management – initial evaluation of a training tool prototype
Source: BMC Med Educ. 2024 Jul 18;24:769. doi: 10.1186/s12909-024-05764-w (PMC11264734; doi:10.1186/s12909-024-05764-w)
Supplement: Supplementary file 1 — Supplementary Material 1 [file 12909_2024_5764_MOESM1_ESM.docx]

Supplement for Immersive Virtual Reality for interdisciplinary trauma management – Evaluation of a training tool prototype

**Supplement 1:** Translation of the questionnaire used, the gray filled numbers are part of the immersion rating scale by Nichols et al [reference 19 in main manuscript]

| **No.** | **Question** | **Answer options** |
| --- | --- | --- |
| 1 | How old are you? | In years |
| 2 | What gender do you assign to yourself? | m / f / d |
| 3 | Do you wear corrective lenses? | y / n |
| 4 | If you chose yes in 3, did you were your corrective lenses during training? | y / n |
| 5 | In which year of your professional occupation are you? | In years |
| 6 | Have you completed residency? | y / n |
| 7 | Did you complete emergency medicine training? | y / n |
| 8 | Have you completed a trauma room training prior to this training? | y / n |
| 9 | How confident did you feel concerning trauma room management prior to the training? | Likert scale (1 to 7, 1 = very much, 7 = not at all) |
| 10 | How confident do you feel concerning trauma room management after training? | Likert scale (1 to 7, 1 = very much, 7 = not at all) |
| 11 | Have you ever used VR equipment in the past? | 1. Never 2. Less than 5 times 3. More than 5 times 4. More than 10 times |
| 12 | Have you felt nauseous, dizzy or unwell or did you fell extensive eyestrain during the training? | Scale 0 – 20 (0 = none, 20 = severe nausea or eyestrain) |
| 13 | In the computer generated world I had the sense of “being there”… | Likert scale (1 to 7, 1 = not at all, 7 = very much) |
| 14 | During the game, how often did you think of the other person(s) in the room with you? | Likert scale (1 to 7, 1 = not at all, 7 = all the time) |
| 15 | How flat and missing in depth did the game appear? | Likert scale (1 to 7, 1 = not at all, 7 = very much) |
| 16 | Do you think of the computer-generated world as… | Likert scale (1 to 7, 1 = something that I saw…, 7 = something that I visit) |
| 17 | How much more enjoyable would it have been to use the game with no-one else in the room? | Likert scale (1 to 7, 1 = no more enjoyable …, 7 = A great deal more enjoyable |
| 18 | How disturbing was the lag or delay between your movements of the controls and the response in the computergenerated world | Likert scale (1 to 7, 1 = didn’t notice it, 7 = completely off-putting) |
| 19 | Whilst you used the game, music played in the background. How much attention did you pay to it? | Likert scale (1 to 7, 1 = none at all, 7 = a great deal) |
| 20 | The computer generated world became more real or present to me compared to the “real world” | Likert scale (1 to 7, 1 = at no time, 7 = almost all the time) |
| 21 | How exhilarated did you feel after the experience? | Likert scale (1 to 7, 1 = felt normal, 7 = felt really exhilirated) |
| 22 | Do you feel like VR is a useful supplement for medical students and medical professionals? | Likert scale (1 to 7, 1 = very much, 7 = not at all) |
| 23 | Do you have any notes? | Free form |

**Supplement 2a**: table of participants’ characteristics sorted by specialist training

| **Parameter** | **Residents** | **Specialists** |
| --- | --- | --- |
| n | 21 | 10 |
| m / f / d | 12 / 9 / 0 | 6 / 4 / 0 |
| Age (median, min.; max. in years) | 31 (26; 36) | 36 (32; 58) |
| Years working in anesthesiology (median, min.; max.) | 2 (1; 6) | 8 (6; 32) |
| Emergency medicine training completed (y / n) | 4 / 17 | 10 / 0 |
| PGY 3 or younger | 14 | 0 |
| Prior experience with VR technology (none / less than 5 times / more than 5 times / more than 10 times) | 14 / 6 / 0 / 1 | 5 / 3 / 1 / 1 |
| Visual aid (y / n) | 13 / 8 | 6 / 4 |
| Visual aid used (y / n) | 9 / 4 | 4 / 2 |
| Emergency trauma room training completed in the past (y / n) | (6 / 15) | (7 / 3) |

**Supplement 2b:** a table of participants characteristics.

| **Parameter** | **All** | **Residents** | **Specialists** |
| --- | --- | --- | --- |
| n | 31 | 21 | 10 |
| m / f / d | 18 / 13 / 0 | 12 / 9 / 0 | 6 / 4 / 0 |
| Age (median, min.; max. in years) | 33 (26; 58) | 31 (26; 36) | 36 (32; 58) |
| Years working in anesthesiology (median, min.; max.) | 4 (1; 25) | 2 (1; 6) | 8 (6; 32) |
| Emergency medicine training completed (y / n) | 14 / 17 | 4 / 17 | 10 / 0 |
| PGY 3 or younger | 14 | 14 | 0 |
| Prior experience with VR technology (none / less than 5 times / more than 5 times / more than 10 times) | 19 / 9 / 1 / 2 | 14 / 6 / 0 / 1 | 5 / 3 / 1 / 1 |
| Visual aid (y / n) | 19 / 12 | 13 / 8 | 6 / 4 |
| Visual aid used (y / n) | 13 / 6 | 9 / 4 | 4 / 2 |
| Emergency trauma room training completed in the past (y / n) | 13 / 18 | (6 / 15) | (7 / 3) |

**Supplement 3:** Detailed script of the scenarios.

Scenario 1 – trauma patient

Scene 1: The trauma leader is in the emergency trauma room with a student intern, who is shadowing him. He trauma leader explains the surroundings to the student, when he receives a call from the emergency dispatcher: a young patient has been involved in a traffic accident and is brought to the emergency trauma room because of severe abdominal and thoracic pain and bruise marks indicating more severe trauma. Vital signs are stable at the time of reporting. The trauma leader now must inform the second anesthetist, the anesthesiology nurse and ICU as well as order RPCs to the trauma room, which involves several phone calls.

Scene 2: The trauma surgeon is in the emergency trauma room and informs all other member of the trauma team of the patient arriving. This includes a trauma nurse, the radiologist, and the abdominal surgeon on duty.

Scene 3: The trauma team meets in the emergency trauma team. The trauma leader, who is now wearing a high visibility vest indicating his function as trauma leader, briefs everyone on the currently available information and everybody’s tasks as well as goes over questions.

Scene 4: Everybody is wearing protective gear (gloves, face masks, googles and protective gowns) when the patient arrives. The emergency doctor gives a brief handover. Since the patient’s vital signs are still stable, the collective decision is made to skip FAST and go for a CT scan.

Scene 5: The viewer is already in the CT room with the radiology technician, who is preparing for the patient, who is about to arrive. She turns on the heating lamp above the stretcher and opens doors. Shortly after, patient and trauma team arrive. The patient is transferred to the CT and while anesthesiology is busy with monitoring, trauma surgeon and abdominal surgeon do quick assessments of the patient. Colleagues from ICU have arrived as well for further assessment of the patient.

Scene 6: The team is now in the department of radiology with view of the trauma CT. All available findings of clinical assessment, blood gas analysis and monitoring are summarized by the trauma leader.

Scene 7: The patient is transferred back to the stretcher and log roll is performed in order to examine his backside.

Scene 8: Trauma leader, trauma surgeon and abdominal surgeon are at the radiologist’s office and discuss the findings of the CT scan. Luckily there are no severe injuries, thus emergency trauma room is resolved.

Scenario 2:

Scene 1: The trauma leader is in the emergency trauma room with a student intern, who is shadowing him. He trauma leader explains the surroundings to the student, when he receives a call from the emergency dispatcher: an elderly patient was found without any observed trauma. He is unresponsive and is vital signs show hypotonia and tachycardia. The trauma leader now must inform the second anesthetist, the anesthesiology nurse and ICU as well as order RPCs to the trauma room, which involves several phone calls.

Scene 2: The trauma surgeon is in the emergency trauma room and informs all other member of the trauma team of the patient arriving. This includes a trauma nurse, the radiologist, and the abdominal surgeon on duty.

Scene 3: The trauma team meets in the emergency trauma team. The trauma leader, who is now wearing a high visibility vest indicating his function as trauma leader, briefs everyone on the currently available information and everybody’s tasks as well as goes over questions.

Scene 4: Everybody is wearing protective gear (gloves, face masks, googles and protective gowns) when the patient arrives. The emergency doctor gives a brief handover. The patient is intubated and in a compensated state under high pressure iv fluid therapy. FAST shows free fluid in the abdominal cavity. Since there is no observed trauma and the patient is in a compensated state, the collective decision is made to transfer the patient to the CT.

Scene 5: The viewer is already in the CT room with the radiology technician, who is preparing for the patient, who is about to arrive. She turns on the heating lamp above the stretcher and opens doors. Shortly after, patient and trauma team arrive. The patient is transferred to the CT and while anesthesiology is busy with monitoring, trauma surgeon and abdominal surgeon do quick assessments of the patient. Colleagues from ICU have arrived as well for further assessment of the patient.

Scene 6: The team is now in the department of radiology with view of the trauma CT. All available findings of clinical assessment, blood gas analysis and monitoring are summarized by the trauma leader.

Scene 7: Trauma leader, trauma surgeon and abdominal surgeon are at the radiologist’s office and discuss the findings of the CT scan. The patient shows a lacerated spleen with active bleeding and is transferred to angiography for interventional, organ-sparing treatment. The emergency trauma room is resolved.

**Supplement 4:** Participants characteristics sorted by VR experience.

| **Parameter** | **Any VR experience** | **No VR experience** | **p** |
| --- | --- | --- | --- |
| n | 12 | 19 |  |
| m / f / d | 10 / 2 / 0 | 8 / 11 / 0 | **0.032** |
| Age (in years, median, min.; max.) | 32 (27; 42) | 33 (26; 58) | 0.914 |
| VIMS (median, min.; max.) | 0 (0; 2) | 2 (0; 13) | **0.031** |
| Depth perception (median, min.; max.) | 1.5 (1; 4) | 2 (1; 6) | 0.322 |
| VR useful for training (median, min.; max.) | 1 (1; 2) | 1 (1; 2) | 0.938 |
| Exhilaration (median, min.; max.) | 1 (1; 3) | 1 (1; 5) | 0.305 |

The table shows a comparison of different metrics by VR experience. Due to the small sample size, all VR experience is condensed as “any VR experience”. There are no significant differences in age, perception of depth, exhilaration and regarding VR useful for training. It is noticeable that significantly more men than women had prior experience with VR technologies. There is also a significant difference in VIMS, however on a very low level, which is to be considered not clinically relevant.
